# Supplementary material for: Piezo2 regulates colonic mechanical sensitivity in a sex specific manner in mice
Source: Nat Commun. 2023 Apr 15;14:2158. doi: 10.1038/s41467-023-37683-7 (PMC10105732; doi:10.1038/s41467-023-37683-7)
Supplement: Supplementary file 1 — Supplementary Information [file 41467_2023_37683_MOESM1_ESM.pdf]

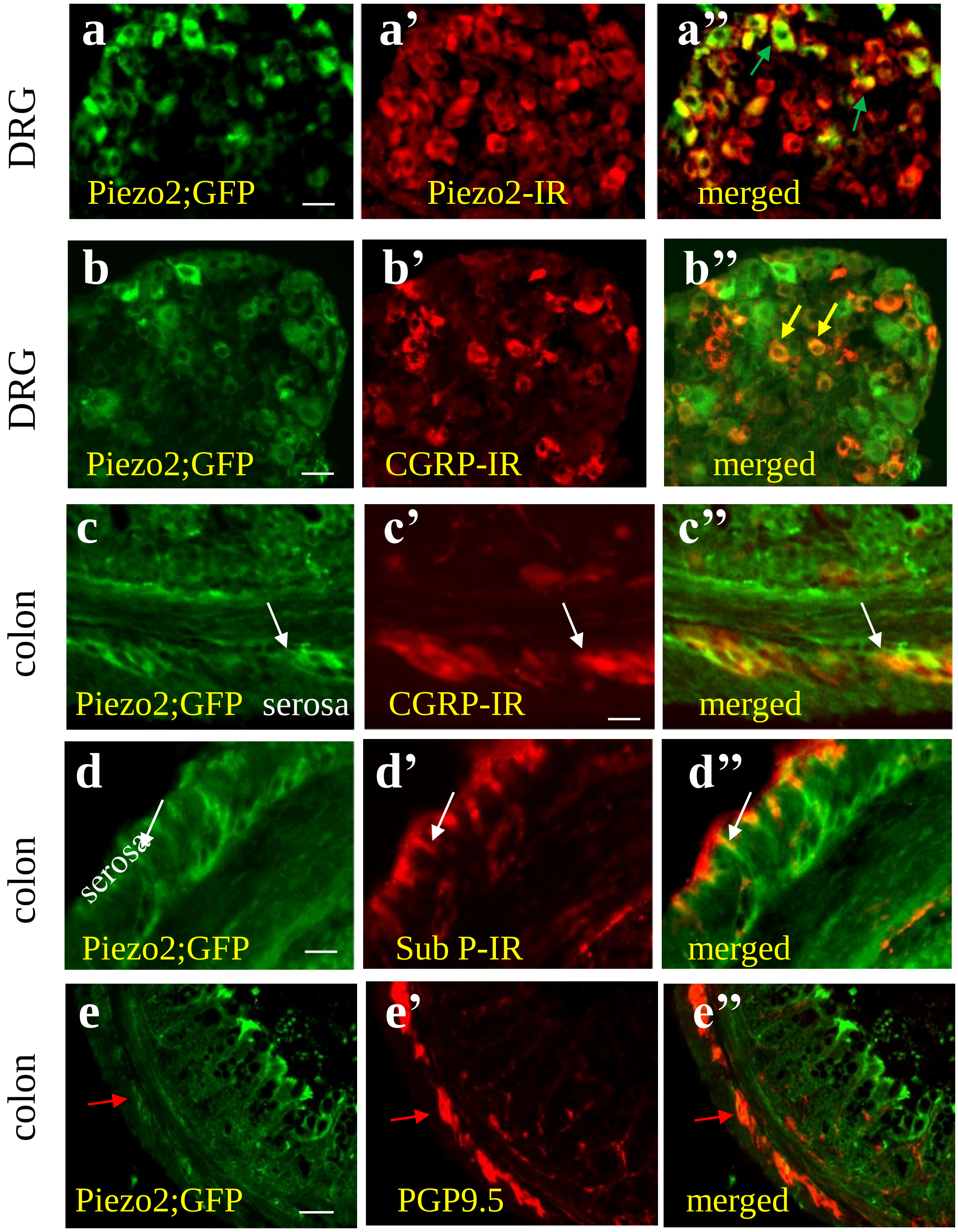


Figure S1. **Piezo2;GFP mice reveals Piezo2 distribution in DRG and the distal colon.** (a-a''): Piezo2;GFP (a: green fluorescent protein) and Piezo2-immunoreactivity (a': IR) are largely co-localized in dorsal root ganglia (a'': DRG). Scale bar: 50 µm. (b-b''): Piezo2;GFP (b: green cells) partially co-expresses with calcitonin gene-related peptide (b': CGRP) in DRG (b'': yellow cells show co-localization). Scale bar: 50 µm. (c-c''): Piezo2;GFP (c: green stain) is present in CGRP fibers (c': red stain) in the distal colon (c'': yellow stain shows co-localization). Scale bar: 500 µm. (d-d''): Piezo2;GFP (d: green stain) is present in substance (Sub) P fibers (d': red stain) in the distal colon (d'': yellow stain shows co-localization). Scale bar: 300 µm. (e-e''): Piezo2;GFP (e: green stain) is present in the enteric nervous plexus visualized by protein gene product 9.5 (PGP9.5) immunoreactivity (e': red stain) in the distal colon (e'': merge shows co-localization) . Scale bar: 500 µm. The same animals that are used for Figure 1c are further assessed for Piezo2-IR (a) and CGRP-IR (b) of DRG sections, and for CGRP-IR (c), Sub P-IR (d) and PGP9.5-IR (e) of colon sections.

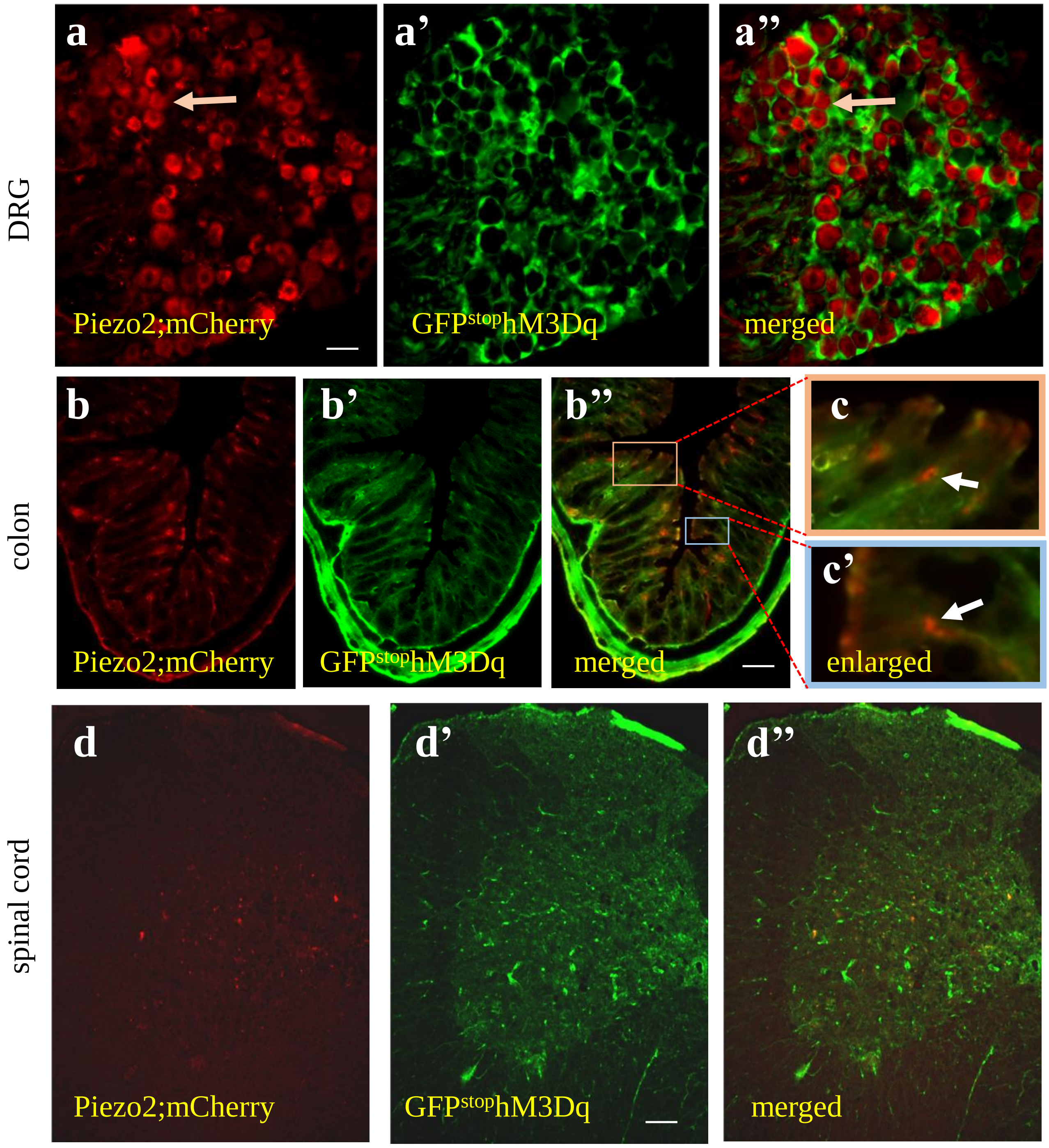


Figure S2. **Piezo2;hM3Dq mice reveals Piezo2 expression in DRG neurons, EC cells in the distal colon but not in the spinal cord.** (a-a"): Piezo2;mCherry (a: red cells), contrasted by a green fluorescent protein (GFP) background (a': green dye), appears in dorsal root ganglia (DRG) neurons (a": red cells are Piezo2-expressing neurons. 3 male and 2 female mice are evaluated, also see Figure 1d and Figure 2e and 2g). Scale bar: 50 µm. (b-b"): Piezo2;mCherry (b: red stain) expresses in a variety of regions in the distal colon (b': GFP background; b'': merge from b and b'. The number of Piezo2;mCherry mice used for this assessment refers to Figure 1e). Scale bar: 500 µm. (c-c'): Zooming-in Piezo2;mCherry expression in colonic mucosal cells morphologically resembling enterochromaffin (EC) cells. (d-d"): Piezo2;mCherry (d: red stain) is scarce in the spinal cord (d': GFP background; d": merge from d and d'. The number of Piezo2;mCherry mice used for this assessment refers to Figure 1f). Scale bar: 100 µm.

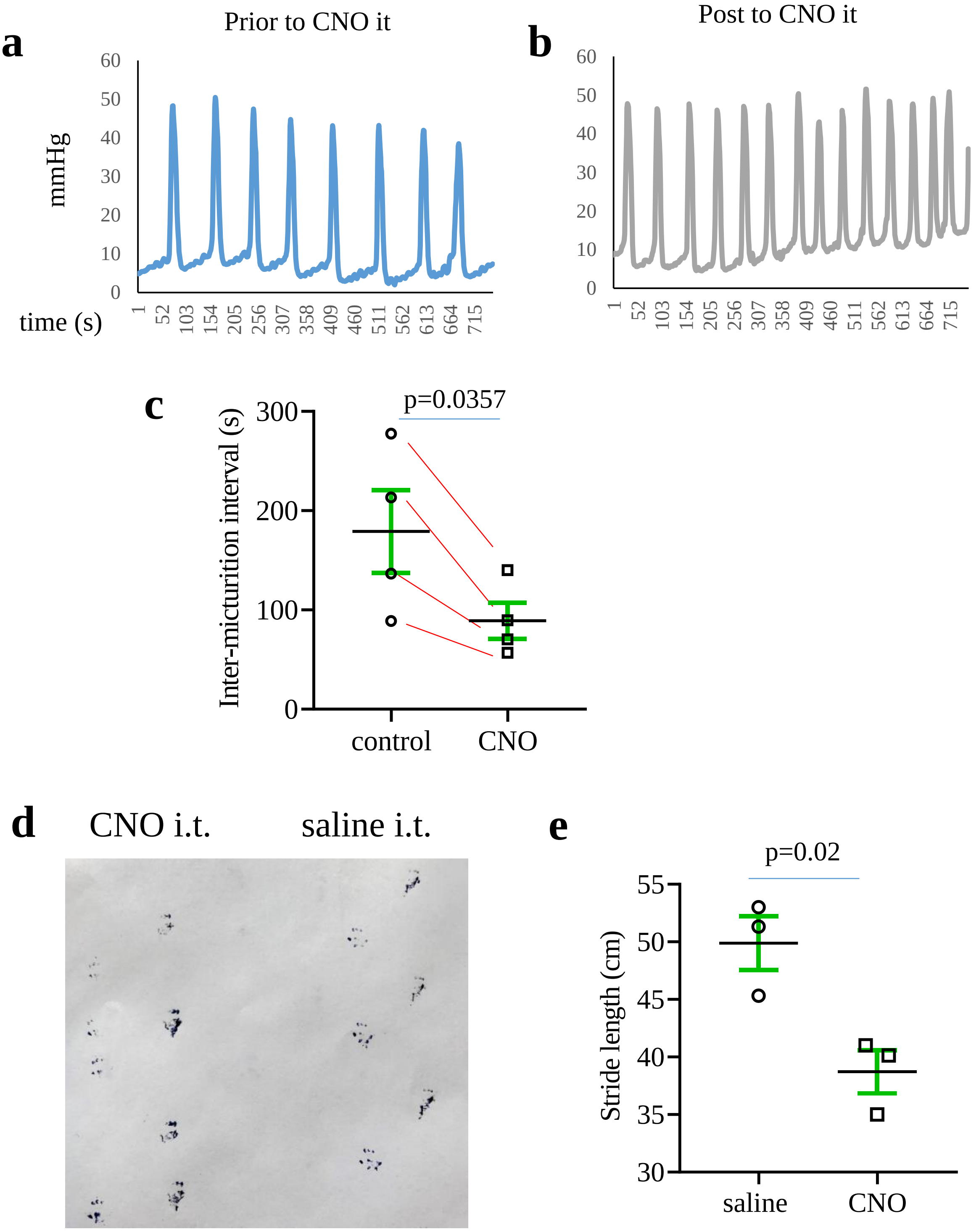


Figure S3. **Effects of intrathecal chemogenetic activation of Piezo2;hM3Dq mice on the urinary bladder and sensory activity.** (a): Micturition recording prior to clozapine N-oxide (CNO) injection. (b): Micturition recording post CNO injection. (c): Inter-micturition interval analysis. n=4 biologically independent male mice. Data are presented as mean values +/- SEM. Two-tailed paired *t* test (p=0.0357, t=3.641). (d): Gait analysis of male mice. (e): Stride length. n=3 biologically independent mice. Data are presented as mean values +/- SEM. Two-tailed unpaired *t* test (p=0.0201, t=3.743, F=1.548).

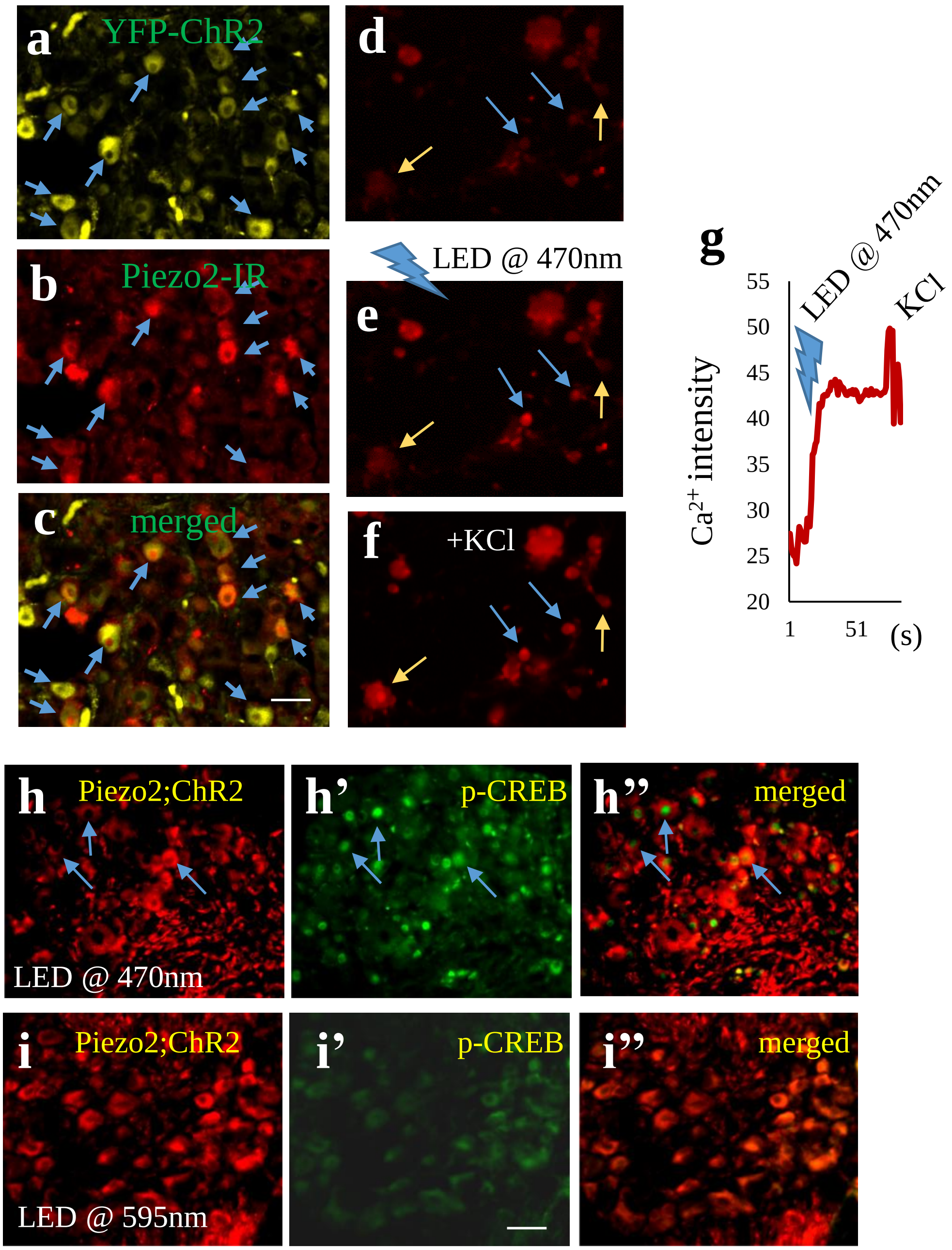


Figure S4. **Optogenetic activation of Piezo2-expressing DRG neurons.** (a): Injection of adeno-associated viruses (AAV) carrying ChR2 (Channelrhodopsin-2)/YFP (yellow fluorescent protein) into Piezo2-Cre mice (3 mice are injected) results in ChR2;YFP expression in dorsal root ganglia (DRG) neurons. (b): Piezo2 immunoreactivity (IR) in DRG (one DRG from each animal is tested). (c): Merged photograph to show co-localization (orange cells indicated by blue arrows) of Piezo2-IR and YFP-ChR2. Representative section is from lumbar L1 DRG. (d): Cultured Piezo2;ChR2 DRG neurons are loaded with calcium ($Ca^{2+}$) indicator RhoD4-AM for optogenetic imaging. (e): Five pulses of blue light stimulations (a total of 11 wells are tested for different blue light stimulation paradigm to customize for optimal photostimulation conditions) activate Piezo2-expressing DRG neurons due to their expression of ChR2 (blue arrows indicate photostimulation-activated cells). (f): Enhanced $Ca^{2+}$ activity in DRG neurons post potassium chloride (KCl) treatment (100 mM) as positive control (yellow arrows indicate cells that are activated by KCl but not LED). (g): $Ca^{2+}$ transients in DRG neurons. (h-h”): Piezo2;ChR2 DRG explants are stimulated by above customized optimal condition of blue light @ 470nm stimulation (h) which increases the phosphorylation of cAMP response element-binding protein (h’: p-CREB) in Piezo2-expressing DRG neurons (h”: merge from h and h’) when compared to negative (sham) non-photostimulation (i-i”) (2 pairs of DRGs are tested to confirm the effectiveness of blue light stimulation). Scale bar: 50 µm.

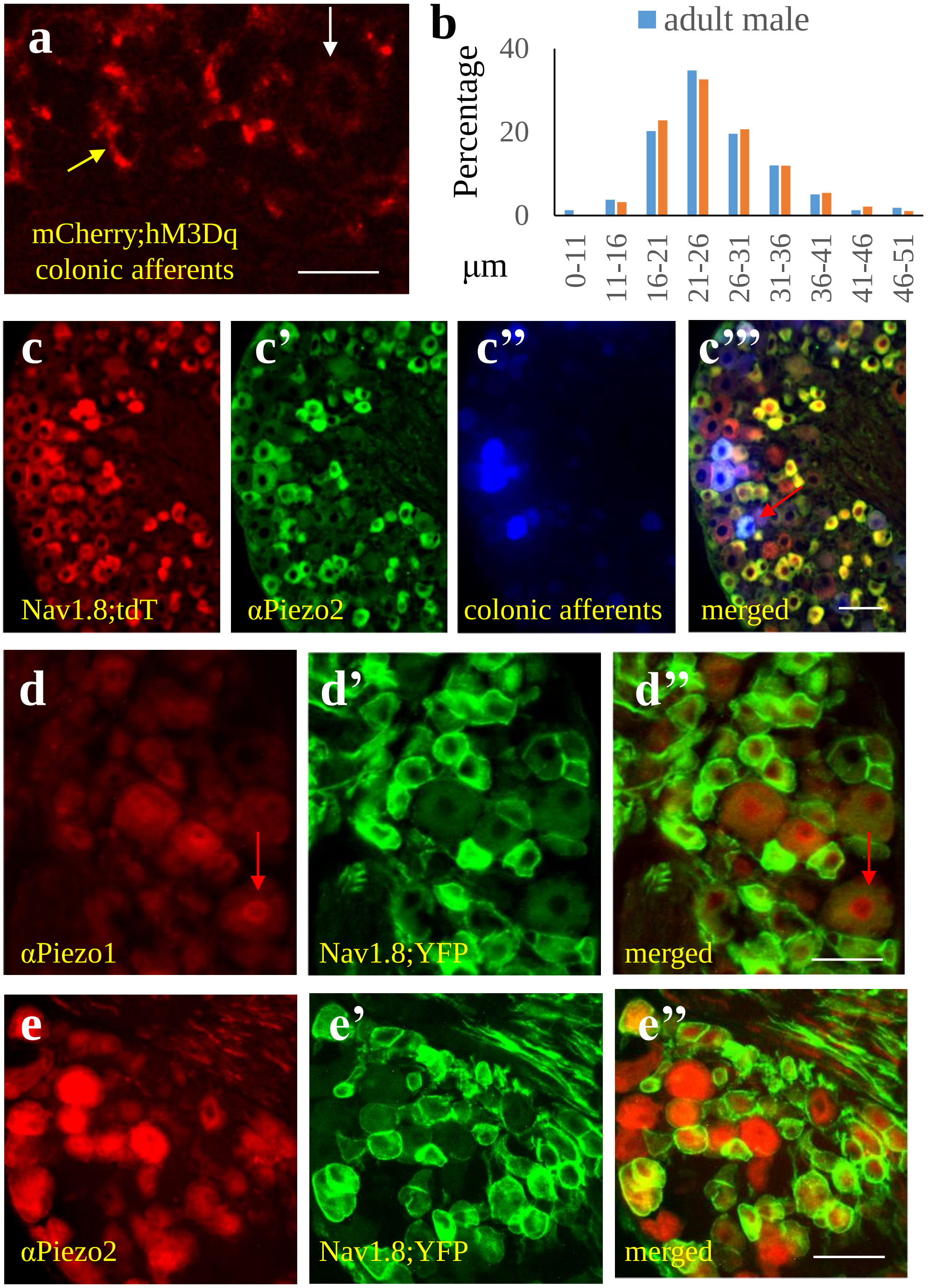


Figure S5. **Piezo2 and Piezo1 expression in DRG.** (a): Retrograde adeno-associated viruses (AAV)-mCherry;hM3Dq labels Piezo2-expressing colonic afferent neurons in dorsal root ganglia (DRG, representative microphotograph is from a L2 DRG) (a female mouse is randomly chosen for DRG sections at every spinal levels from T13-L6, and a thoracolumbar DRG from one additional male and female are assessed for mCherry expression). (b): The percentage of Piezo2-expressing DRG neurons distributes to each category defined by diameters of cell size in adult male and newborn (postnatal day 8) male mice as comparison. (c-c’’’): Co-expression of Nav1.8;tdTomato (c: red cells from 3 mice) and Piezo2 immunoreactivity (IR: c’: green cells) in colonic afferent neurons (c”: blue cells) that are merged to show co-localization (c’’’: arrow indicates co-expression). Scale bar: 50 µm. (d-d”): Co-expression of Piezo1 (d: red cells) and Nav1.8 (d’: green cells) in DRG neurons (d”: arrow indicates Piezo1-immunoreactivity (IR)). Scale bar: 50 µm. (e-e”): Co-expression of Piezo2 (e: red cells) and Nav1.8 (e’: green cells) in the same sets of DRG sections that are also used for Piezo1 expression as comparison. The number of mice evaluated in (d-d”) and (e-e”) refers to Figure 4b and 4c. Scale bar: 50 µm.

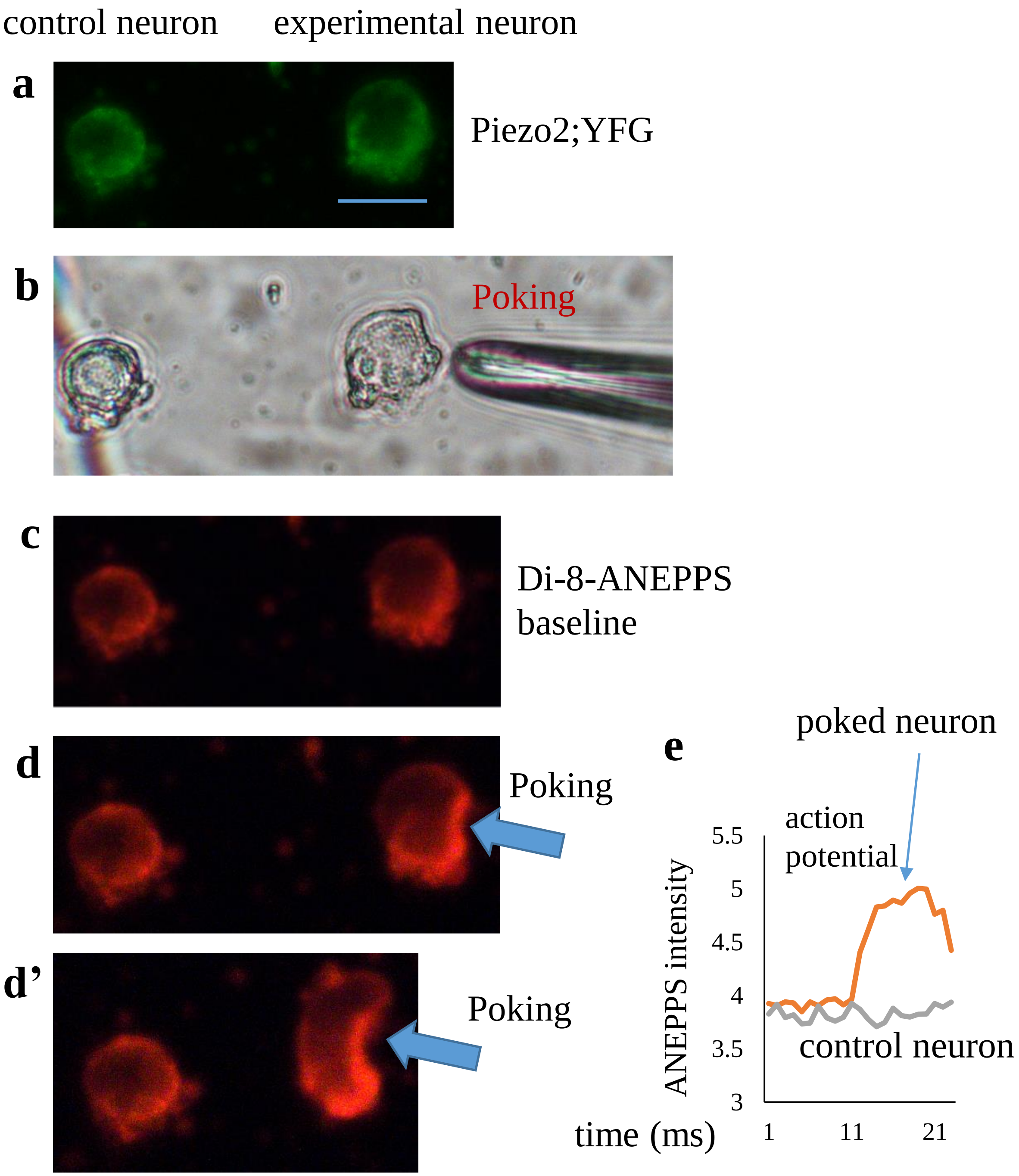


Figure S6. **Piezo2-expressing DRG neurons are subject to glass pipette poking.** (a): Two Piezo2;YFP (yellow fluorescent protein)-expressing dorsal root ganglia (DRG) neurons in the same microscopic field. (b): One of the Piezo2;YFP neuron is stimulated by a glass pipette tip through poking. (c): Baseline intensity of Di-8-ANEPPS. (d-d’): Increment in the distance of poking elicits an increase in Di-8-ANEPPS intensity. (e): Plotted voltage change curves in the poked and control neurons. A total of 20 Piezo2;YFP expressing neurons from 2 male mice and 7 Piezo2;YFP-expressing neurons from 1 female mice are successfully poked and all of them show a certain degree of activation. Scale bar: 50 µm.

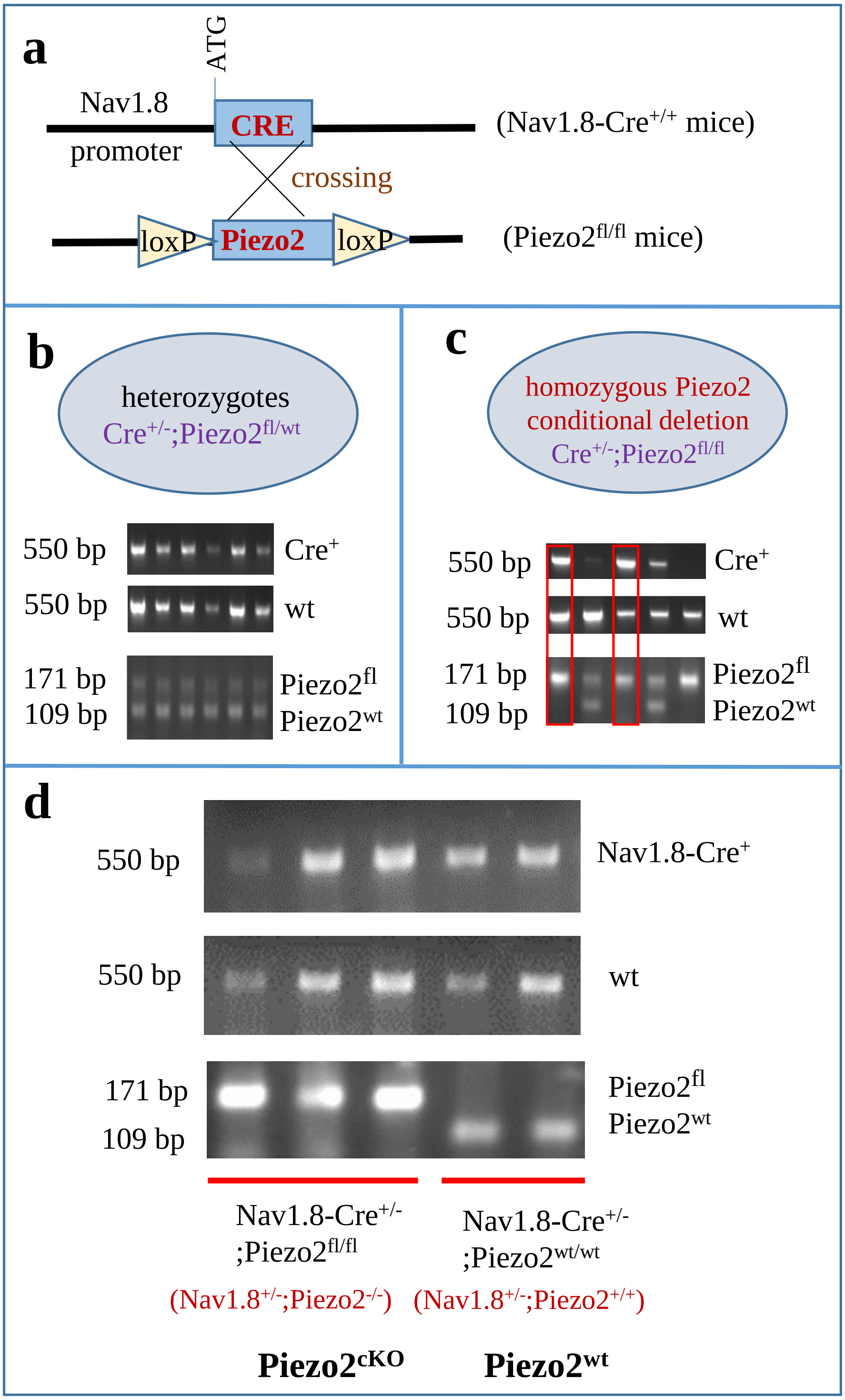


Figure S7. **Strategies of conditional deletion of Piezo2 from nociceptive neurons.** (a): Schematic diagram demonstrates parenting mice that are used for breeding. Nav1.8-$Cre^{+/+}$ mice and floxed Piezo2 ($Piezo2^{fl/fl}$) are used. (b): First generation that produces heterozygous Nav1.8-Cre and Piezo2 (Nav1.8-$Cre^{+/-}$;$Piezo2^{fl/wt}$). Each column is from a single animal. Genotypes of 6 mice are shown here. (c): Further breeding to generate homozygous Piezo2 conditional deletion (Nav1.8-$Cre^{+/-}$;$Piezo2^{fl/fl}$). Among the 5 mice shown here, 2 of them (highlighted by red rectangles) are good for further breeding. (d): Here shows representative 5 mice that include 3 mice with $Piezo2^{cKO}$ (Nav1.8-$Cre^{+/-}$;$Piezo2^{fl/fl}$) and 2 mice with $Piezo2^{wt}$ (Nav1.8-$Cre^{+/-}$;$Piezo2^{wt/wt}$) to be used for data generation.

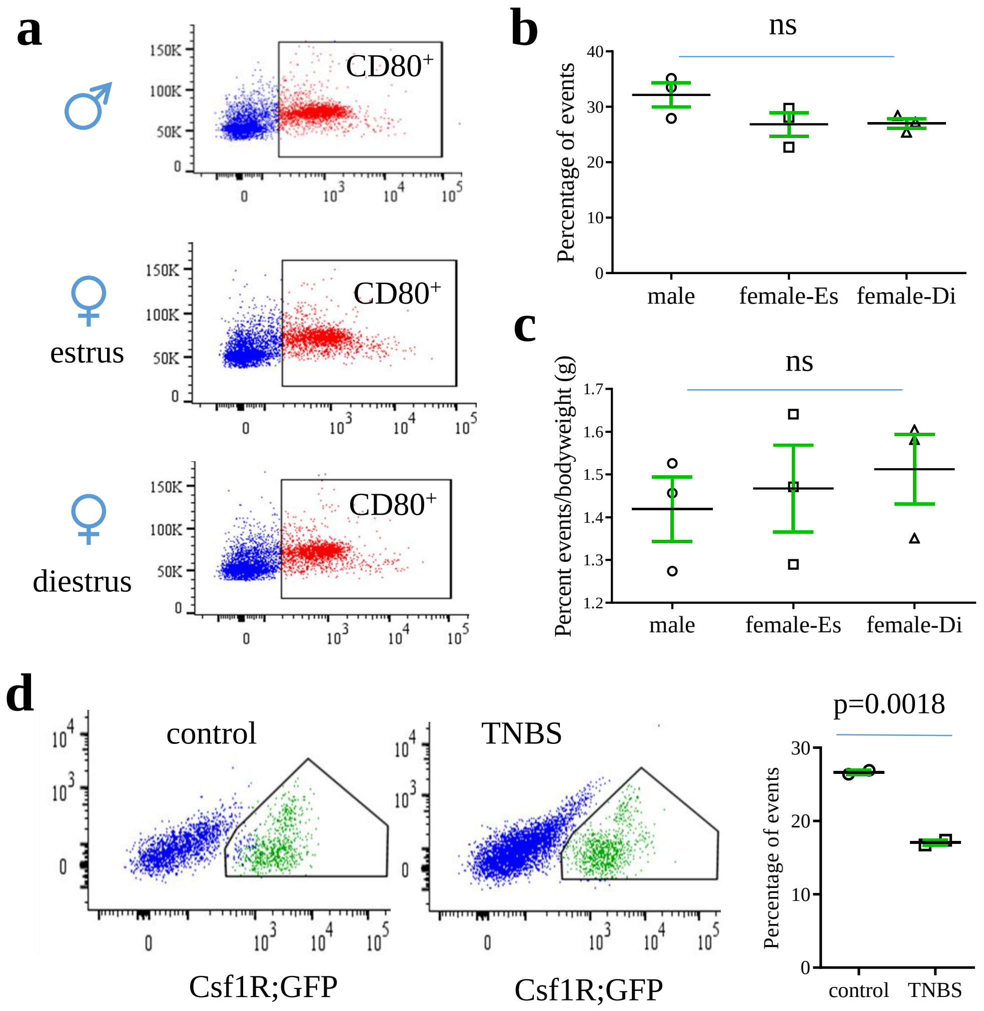


Figure S8. **Macrophage levels in DRG.** (a): The cluster of differentiation 80 positive ($CD80^+$) macrophages in dorsal root ganglia (DRG) of $Piezo2^{wt}$ male, estrus female and diestrus female mice. (b): Quantification of macrophages in DRG of $Piezo2^{wt}$ male (n=3), estrus female (female-Es, n=3) and diestrus female (female-Di, n=3) biologically independent mice. Data are presented as mean values +/- SEM. One-way ANOVA with Newman-Keuls Multiple Comparison Test. ns: Not significant for all comparison groups (F=2.756). (c): Normalization of macrophage levels with body weight. n=3 biologically independent mice for each group. Data are presented as mean values +/- SEM. One-way ANOVA with Newman-Keuls Multiple Comparison Test. ns: Not significant for all comparison groups (F=0.2917). (d): Macrophages levels in colony stimulating factor 1 receptor (Csf1R) promoter-driven green fluorescent protein (GFP) expressing mice with or without colonic inflammation. n=2 biologically independent mice for each group. Data are presented as mean values +/- SEM. Two-tailed unpaired *t* test (p=0.0018, t=23.7).

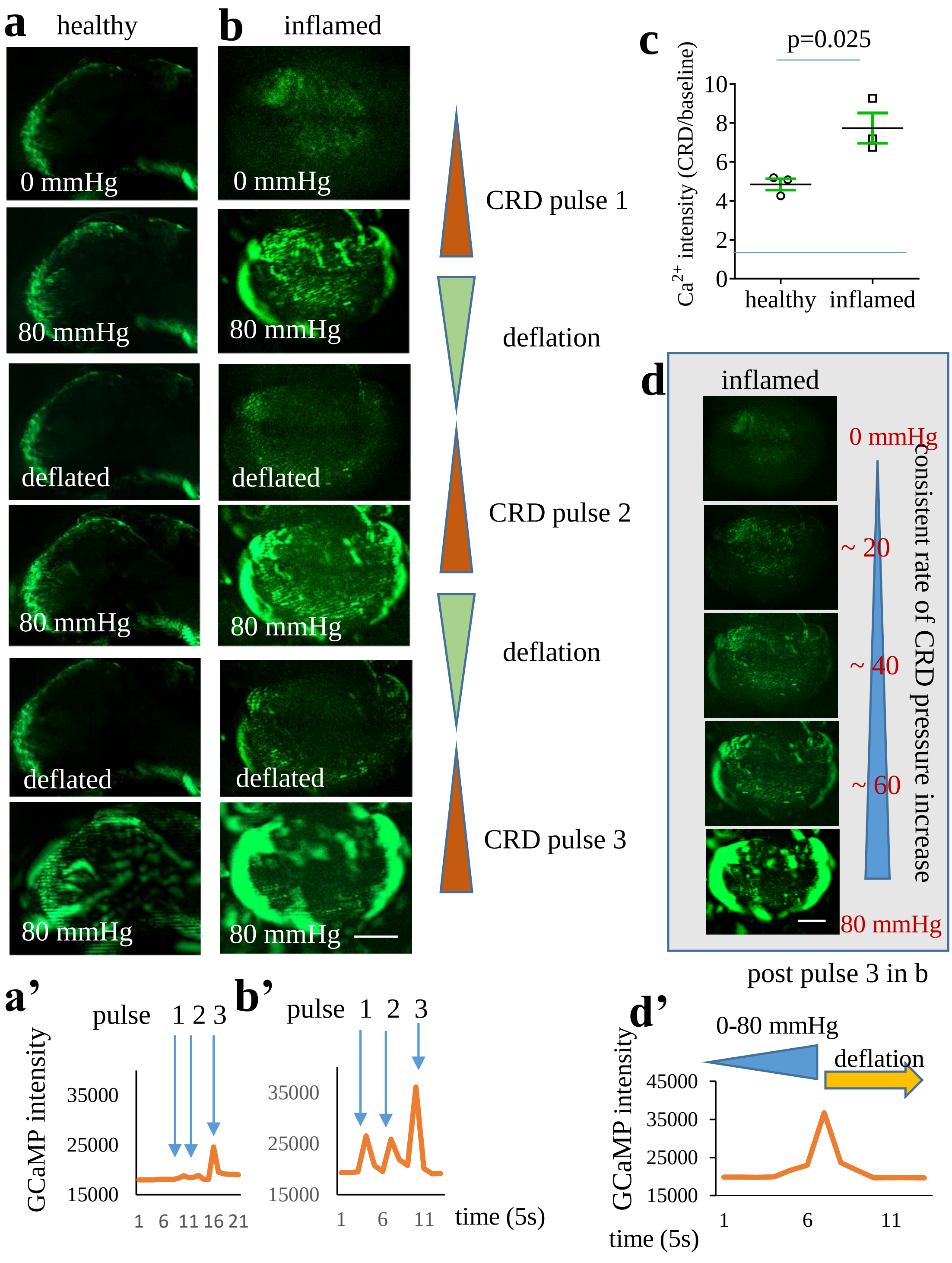


Figure S9. **$Ca^{2+}$ transients in whole DRG in intact mice in response to CRD.** (a): Colorectal distension (CRD) pulses in healthy GCaMP mice (summary data from 3 mice is shown in c). Three pulses with 10-s durations and 5-s intervals are applied which elicits modest calcium ($Ca^{2+}$) transients in dorsal root ganglia (DRG) (a'). (b): CRD pulses in colon inflamed GCaMP mice (summary data from 3 mice is shown in c). Three pulses with 10-s durations and 5-s intervals are applied which elicits larger $Ca^{2+}$ transients in DRG (b') when compared to healthy animals. (c): Fold increases in $Ca^{2+}$ intensity in response to 80 mmHg in comparison to baseline $Ca^{2+}$ levels prior to CRD stimulation for each treatment. n=3 biologically independent mice for each group. Data are presented as mean values +/- SEM. Two-tailed unpaired *t* test (p=0.0252, t=3.486, F=6.958). (d): DRG responses to CRD after 3 pulses of 0-80 mmHg stimulation to validate the aliveness of DRG cells. An increment in the intensity of $Ca^{2+}$ signals is evident (d'). Scale bar: 200 µm.

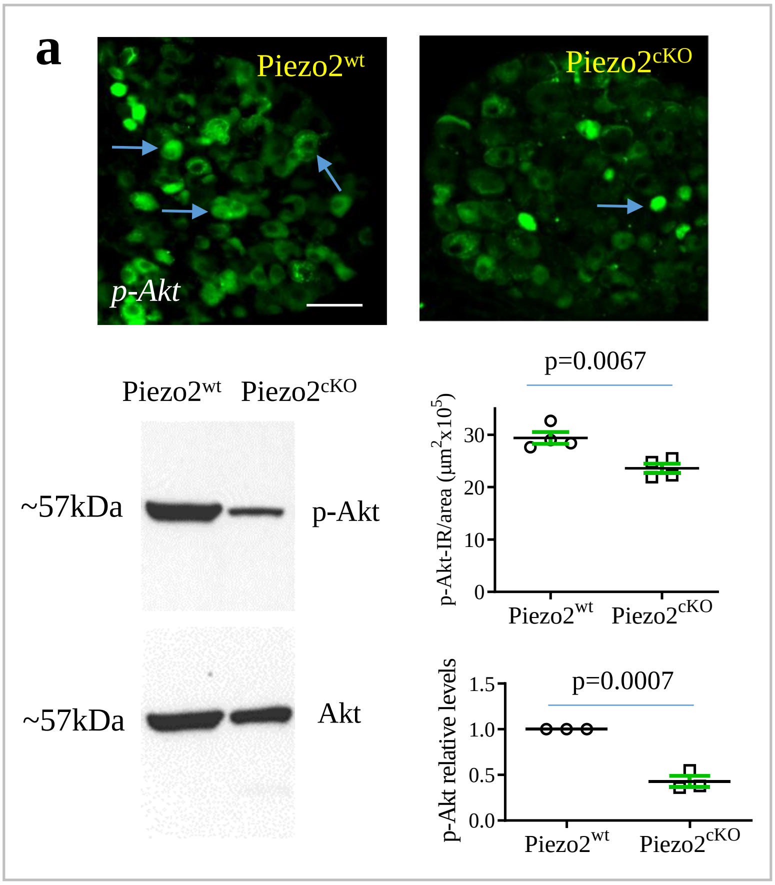


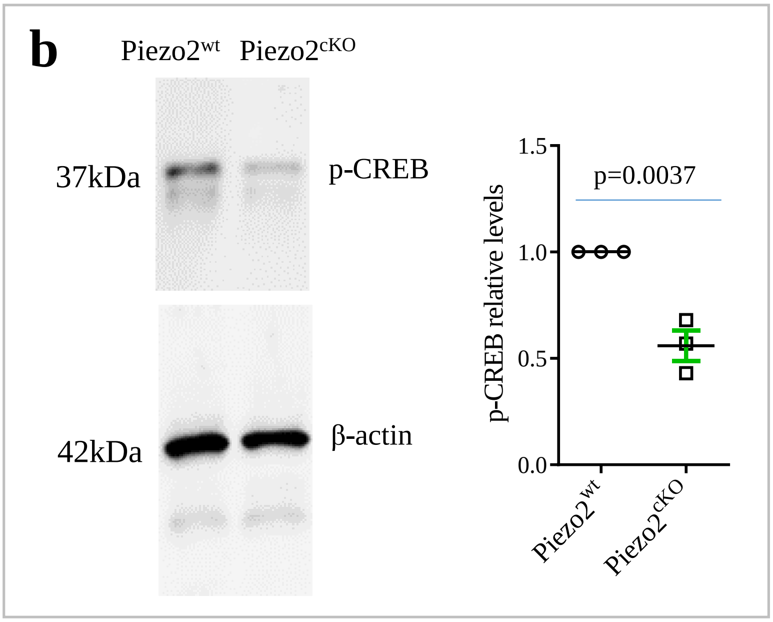


Figure S10. **Effects of noxious CRD on the activity of DRG neurons of Piezo2$^{wt}$ and Piezo2$^{cKO}$ mice.** (a): Immunostaining of phospho-(p)-Akt (representative photographs were from lumbar L1 dorsal root ganglia (DRG), n=4 biologically independent mice for each group. Data are presented as mean values +/- SEM, two-tailed unpaired *t* test, p=0.0067, t=4.050, F=1.576.) and p-Akt western blot (n=3 biologically independent mice for each group. Data are presented as mean values +/- SEM. Two-tailed unpaired *t* test, p=0.0007, t=9.456) demonstrates the effects of Piezo2 deletion on noxious colorectal distension (CRD)-induced Akt activity in thoracolumbar DRG neurons. Scale bar: 50 µm. (b): Western blot of phospho-(p)-CREB (cAMP response element-binding *protein*) demonstrates the effects of Piezo2 deletion on noxious CRD-induced CREB activity in thoracolumbar DRG neurons. n=3 biologically independent mice for each group. Data are presented as mean values +/- SEM. Two-tailed unpaired *t* test, p=0.0037, t=6.082.

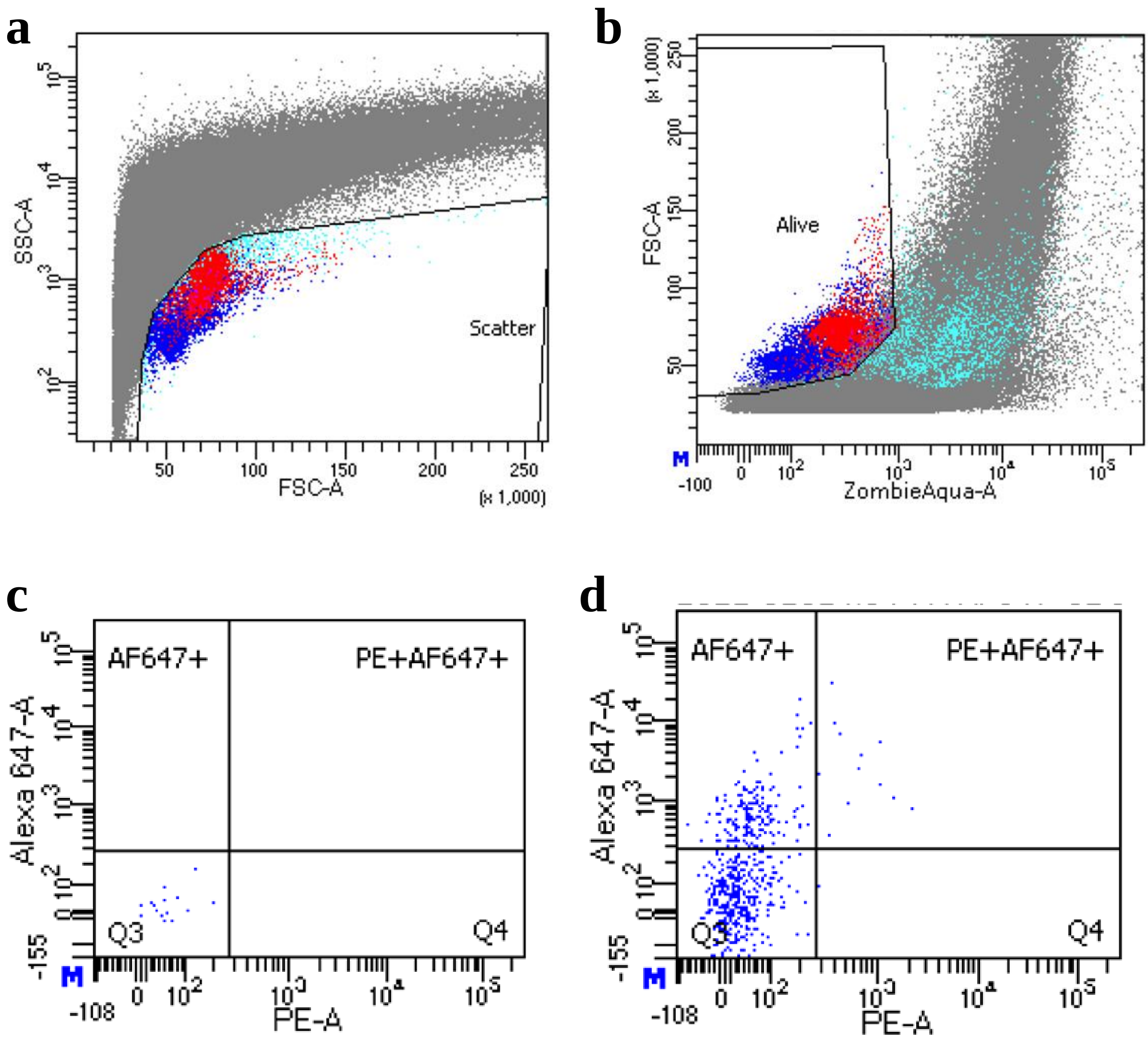


Figure S11: **Flow cytometry gating strategies**. (a) Scatter plot generated using SSC-A versus FSC-A. (b): Selection by using live/dead marker. (c): Representative sample that was not treated with antibody. (d): Representative sample that was treated by specific antibody.

PCR primers:

| | |
|---|---|
| m_Piezo1 F: | GGCTGACAACCCAGCGT |
| m_Piezo1 R: | GGCTACCGTTTTGTCCCAGA |
| m_Piezo2 F: | GTCAATGGTCGCGTGTACCT |
| m_Piezo2 R: | ATGGCGGTAAACGGTGACTT |
| m_CGRP F: | GGGCTCTAGTGTCACTGCTC |
| m_CGRP R: | CCTGTCAAAGGGAGAAGGGT |
| m_β-actin F: | CGCAGCCACTGTCGAGTC |
| m_ β-actin R: | AAGGTCTCAAACATGATCTGGGT |

**Supplementary Table 1**: These primers were used in Figure 4d to characterize the expression of genes of interest.

# Uncropped Figure 4d

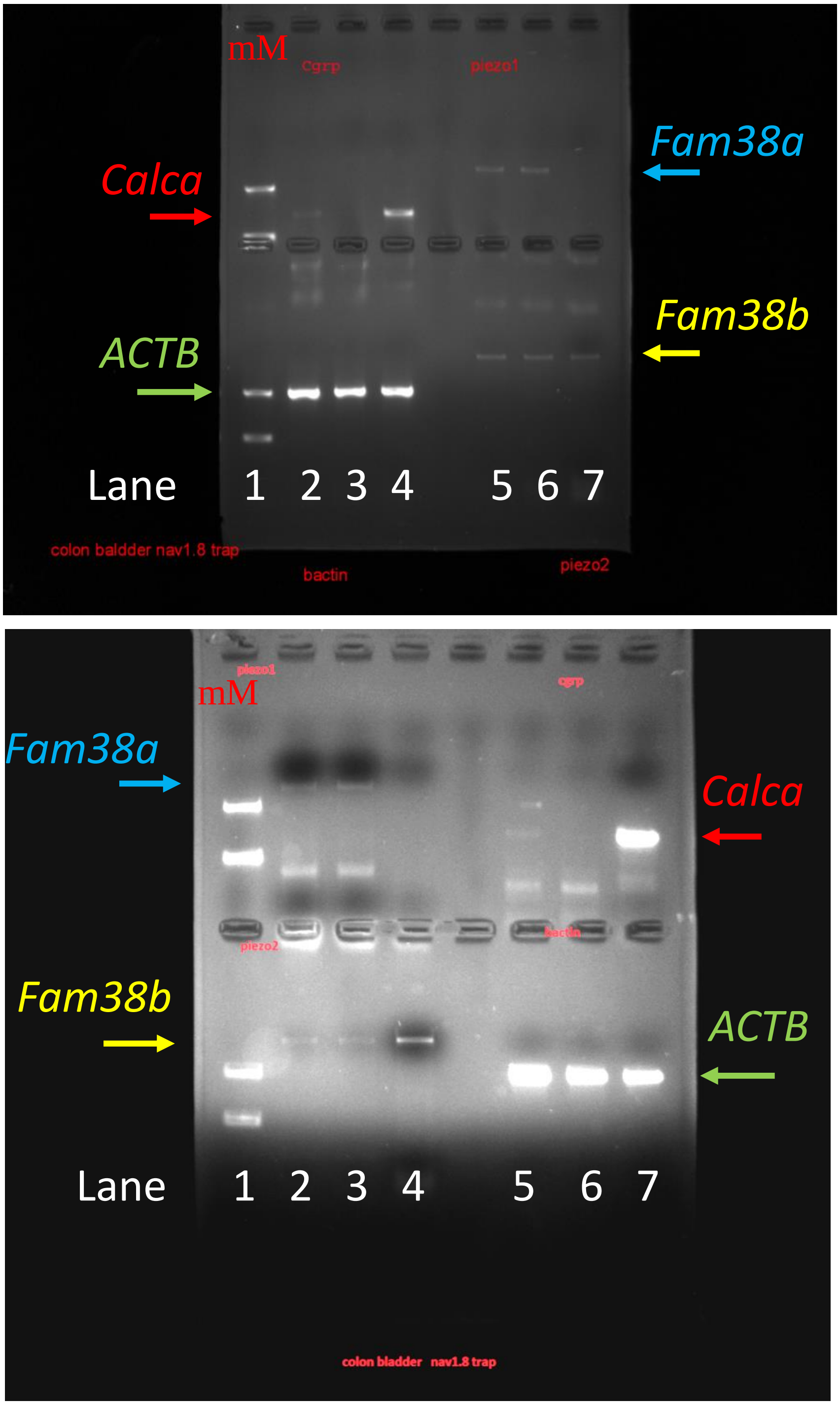


Uncropped agarose gel shows PCR products of mRNA extracted from the distal colon (lanes 2 and 5), urinary bladder (lanes 3 and 6), or nociceptive neurons (lanes 4 and 7). Each gel was products from one animal.
Lane 1: molecular markers (mM: 500, 200 bp)
Genes of interest: *Fam38a* (Piezo1, 508bp), *Calca* (CGRP, 320bp), *Fam38b* (Piezo2, 765bp) and *ACTB* (beta-actin as internal control, 467bp). Primers were listed in Table 1.

# Uncropped Figure S7b

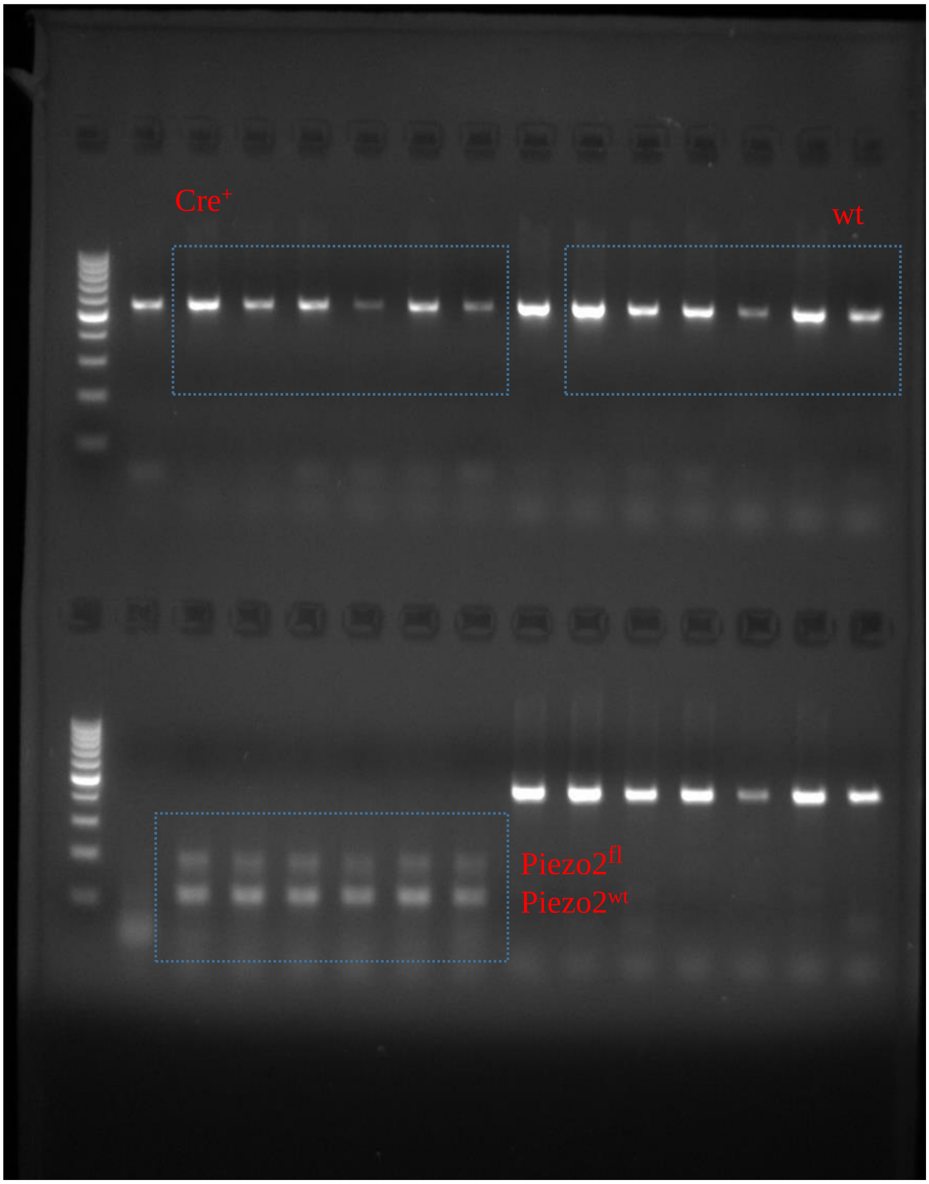


First generation offspring from mating pair Nav1.8-Cre$^{+/+}$ and Piezo2$^{fl/fl}$ demonstrates to contain Nav1.8-Cre$^{+/-}$ and Piezo2$^{fl/wt}$. The highlighted 6 mice are presented in Figure S7b.

# Uncropped Figure S7c

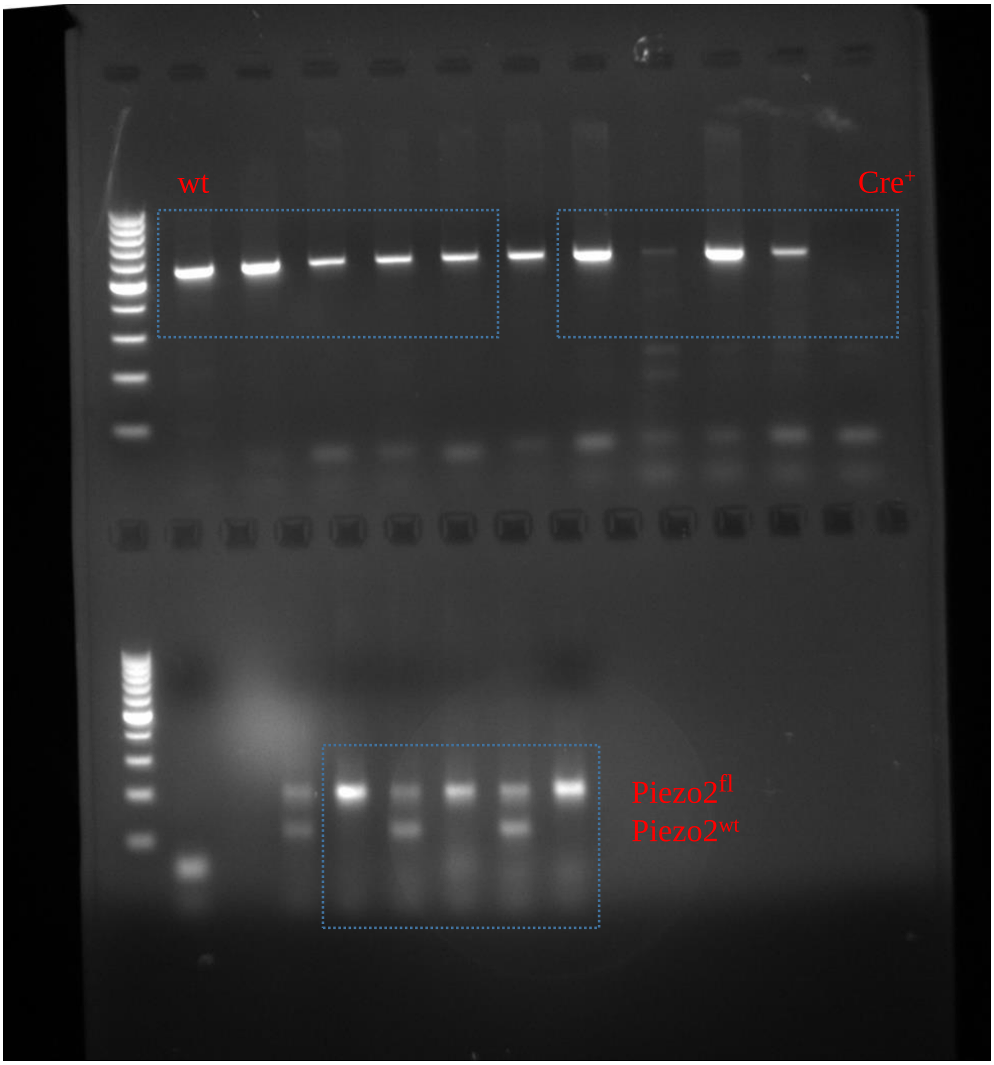


Some of the second generation offspring demonstrates to contain Nav1.8-Cre$^{+/-}$ and Piezo2$^{fl/fl}$, some contain Nav1.8-Cre$^{+/-}$ and Piezo2$^{fl/wt}$ , some Nav1.8-Cre$^{-/-}$ and Piezo2$^{fl/fl}$, and some Nav1.8$^{-/-}$ and Piezo2$^{fl/wt}$ . The highlighted 5 mice are presented in Figure S7c.

# Uncropped Figure S7d

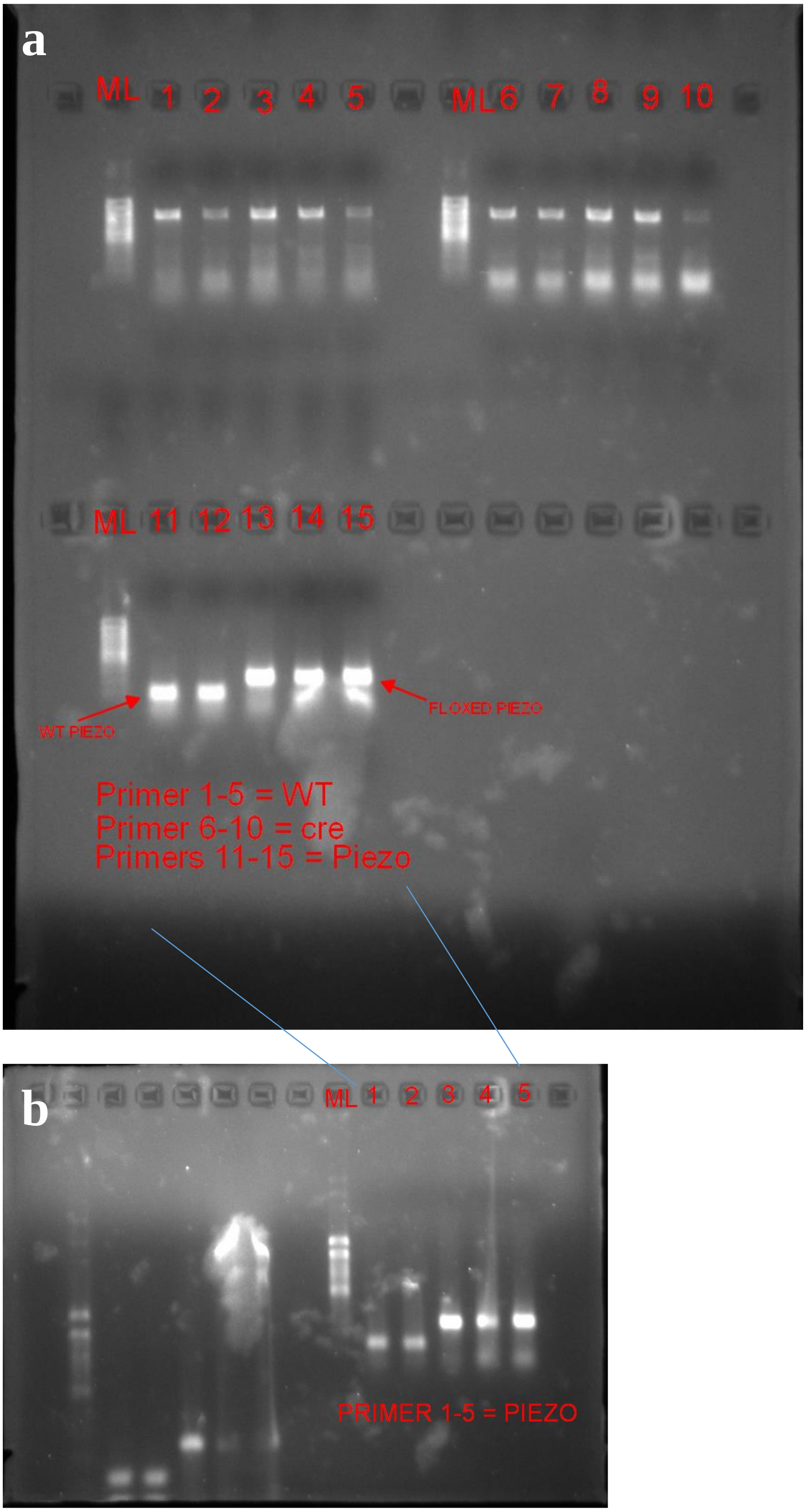


Representative genotying of experimental mice: ML: molecular marker. 5 mice are genotyped. Results show that all 5 mice express one copy of Nav1.8-Cre (Lane 6-10). Two mice (lane 11-12 in gel (a) and lane 1-2 in gel (b) –duplicate for double confirmation) are Piezo2$^{wt}$ (Nav1.8-Cre$^{+/-}$;Piezo2$^{+/+}$) mice. Three mice (lane 13-15 in gel (a) and lane 3-5 in gel (b)) are Piezo2$^{cKO}$ (Nav1.8-Cre$^{+/-}$;Piezo2$^{fl/fl}$) mice. Figure S7d is from these gels with bands horizontally flipped since Piezo2$^{cKO}$ mice are described in the text prior to Piezo2$^{wt}$ mice are mentioned.
