# Supplementary material for: Piezo2 regulates colonic mechanical sensitivity in a sex specific manner in mice
Source: Nat Commun. 2023 Apr 15;14:2158. doi: 10.1038/s41467-023-37683-7 (PMC10105732; doi:10.1038/s41467-023-37683-7)
Supplement: Supplementary file 3 — Reporting Summary [file 41467_2023_37683_MOESM3_ESM.pdf]

nature portfolio

Corresponding author(s): Liya Qiao

Last updated by author(s): Feb 21, 2023

Reporting Summary

Nature Portfolio wishes to improve the reproducibility of the work that we publish. This form provides structure for consistency and transparency in reporting. For further information on Nature Portfolio policies, see our Editorial Policies and the Editorial Policy Checklist.

Statistics

For all statistical analyses, confirm that the following items are present in the figure legend, table legend, main text, or Methods section.

n/a	Confirmed	
☐	☒	The exact sample size (*n*) for each experimental group/condition, given as a discrete number and unit of measurement
☐	☒	A statement on whether measurements were taken from distinct samples or whether the same sample was measured repeatedly
☐	☒	The statistical test(s) used AND whether they are one- or two-sided<br>*Only common tests should be described solely by name; describe more complex techniques in the Methods section.*
☒	☐	A description of all covariates tested
☒	☐	A description of any assumptions or corrections, such as tests of normality and adjustment for multiple comparisons
☐	☒	A full description of the statistical parameters including central tendency (e.g. means) or other basic estimates (e.g. regression coefficient) AND variation (e.g. standard deviation) or associated estimates of uncertainty (e.g. confidence intervals)
☐	☒	For null hypothesis testing, the test statistic (e.g. *F*, *t*, *r*) with confidence intervals, effect sizes, degrees of freedom and *P* value noted<br>*Give P values as exact values whenever suitable.*
☒	☐	For Bayesian analysis, information on the choice of priors and Markov chain Monte Carlo settings
☒	☐	For hierarchical and complex designs, identification of the appropriate level for tests and full reporting of outcomes
☒	☐	Estimates of effect sizes (e.g. Cohen's *d*, Pearson's *r*), indicating how they were calculated

Our web collection on statistics for biologists contains articles on many of the points above.

Software and code

Policy information about availability of computer code

Data collection	Zeiss ZEN pro, Nikon NIS-ELEMENTS-BR, BIO RAD ChemiDoc MP Imaging System with Image Lab Touch, BIO RAD Gel Doc EZ Imager with Image Lab, LabChart 8
Data analysis	Imaging J, LabChart 8, Prism version 5 and 9, FlowJo, Zeiss ZEN pro, Nikon NIS-ELEMENTS-BR.

For manuscripts utilizing custom algorithms or software that are central to the research but not yet described in published literature, software must be made available to editors and reviewers. We strongly encourage code deposition in a community repository (e.g. GitHub). See the Nature Portfolio guidelines for submitting code & software for further information.

Data

Policy information about availability of data

All manuscripts must include a data availability statement. This statement should provide the following information, where applicable:

- Accession codes, unique identifiers, or web links for publicly available datasets
- A description of any restrictions on data availability
- For clinical datasets or third party data, please ensure that the statement adheres to our policy

All data are included in this paper. Source data are provided in an Excel format. Uncropped original gel scans are attached.

Human research participants

Policy information about studies involving human research participants and Sex and Gender in Research.

Reporting on sex and gender	*Use the terms sex (biological attribute) and gender (shaped by social and cultural circumstances) carefully in order to avoid confusing both terms. Indicate if findings apply to only one sex or gender; describe whether sex and gender were considered in study design whether sex and/or gender was determined based on self-reporting or assigned and methods used. Provide in the source data disaggregated sex and gender data where this information has been collected, and consent has been obtained for sharing of individual-level data; provide overall numbers in this Reporting Summary. Please state if this information has not been collected. Report sex- and gender-based analyses where performed, justify reasons for lack of sex- and gender-based analysis.*
Population characteristics	*Describe the covariate-relevant population characteristics of the human research participants (e.g. age, genotypic information, past and current diagnosis and treatment categories). If you filled out the behavioural & social sciences study design questions and have nothing to add here, write "See above."*
Recruitment	*Describe how participants were recruited. Outline any potential self-selection bias or other biases that may be present and how these are likely to impact results.*
Ethics oversight	*Identify the organization(s) that approved the study protocol.*

Note that full information on the approval of the study protocol must also be provided in the manuscript.

Field-specific reporting

Please select the one below that is the best fit for your research. If you are not sure, read the appropriate sections before making your selection.

☒ Life sciences ☐ Behavioural & social sciences ☐ Ecological, evolutionary & environmental sciences

For a reference copy of the document with all sections, see nature.com/documents/nr-reporting-summary-flat.pdf

Life sciences study design

All studies must disclose on these points even when the disclosure is negative.

Sample size	Power analysis was used to determine the sample size to achieve an alpha of 0.05 with a power of 80 %.
Data exclusions	No data was excluded.
Replication	All experiments were performed independently. The number of independent ns are described in the Figure legends. Multiple sections from a single DRG were performed for immunostaining and data were averaged (all raw data were presented in the source data). When replicates of the same sample were needed, we clearly stated them in the paper (i.e., re-run agarose gel to confirm bands in Figure S7d).
Randomization	Animals were randomly divided into experimental groups prior to treatment.
Blinding	Experiments related to cell number counting (immunohistochemistry, calcium imaging) and behavioral analysis were blinded. Animal weighing, western blot, PCR and gel electrophoresis are not blinded, in which the investigators collected and processed samples. The analysis of bands are aided by software.

Reporting for specific materials, systems and methods

We require information from authors about some types of materials, experimental systems and methods used in many studies. Here, indicate whether each material, system or method listed is relevant to your study. If you are not sure if a list item applies to your research, read the appropriate section before selecting a response.

Materials & experimental systems

n/a	Involved in the study	
☐	☒	Antibodies
☒	☐	Eukaryotic cell lines
☒	☐	Palaeontology and archaeology
☐	☒	Animals and other organisms
☒	☐	Clinical data
☒	☐	Dual use research of concern

Methods

n/a	Involved in the study	
☒	☐	ChIP-seq
☐	☒	Flow cytometry
☒	☐	MRI-based neuroimaging

Antibodies

Antibodies used

Immunohistochemistry:
Primary antibodies:
rabbit anti-Piezo2 (1:500, Novus Biologicals LLC, Cat# NBP1-78624)
rabbit anti-CGRP (1:1000, Invitrogen, Cat# PA5-114929)
goat anti-CGRP (1:2000, Abcam, Cat# AB36001)
goat anti-Sub P (1:500, Santa Cruz, Cat# sc-9758)
rabbit anti-PGP9.5 (1:1000, Millipore, Cat# AB1761-I)
rabbit anti-Piezo1 (1:500, Novus Biologicals LLC, Cat# NBP1-78446)
rabbit anti-p-CREB (1:500, Cell Signaling, Cat# 9198)
rabbit anti-p-Akt (1:500, Cell Signaling, Cat# 4046)
Isolectin GS-IB4 (1:500, Molecular Probes, Cat# I21411).

Secondary antibodies:
donkey anti Rabbit (Cy3) (1:500, Jackson Immuno research, Cat# 711-165-152)
donkey anti-goat 594 (1:500, Life Technologies, Cat# A11058)
donkey anti-rabbit 488 (1:500, Life Technologies, A21206)
donkey anti goat 488 (1:500, Life Technologies, Cat# A11055).

Flow cytometry:
Alexa 647-conjugated anti-mouse CD80 (1:200, Biolegend, Cat# 104718, Clone 16-10A1)

Western blot:
Primary antibodies:
rabbit anti-p-Akt (1:1000, Cell Signaling, Cat# 4060)
rabbit anti-Akt (1:1000, Millipore Sigma, Cat# SAB4500800)
rabbit anti-p-CREB (1:1000, Cell Signaling, Cat# 9198)
mouse anti-beta actin (1:5000, Sigma, Cat# A2228).
Secondary antibodies:
anti-rabbit IgG, HRP-linked (1:2500, Cell Signaling, Cat# 7074)
anti-mouse IgG, HRP-linked (1:2500, Cell Signaling, Cat# 7076).

Validation

Primary antibodies that have been validated on the manufacture's websites, publications,or the current study:
rabbit anti-Piezo2 (1:500, Novus Biologicals LLC, Cat# NBP1-78624) (https://www.novusbio.com/products/piezo2-antibody_nbp1-78624; Piezo2-reporter validated: Figure S1a, S4b).
rabbit anti-CGRP (1:1000, Invitrogen, Cat# PA5-114929) (https://www.thermofisher.com/antibody/product/CGRP-Antibody-Polyclonal/PA5-114929)
goat anti-CGRP (1:2000, Abcam, Cat# AB36001) (https://www.abcam.com/products/primary-antibodies/cgrp-antibody-ab36001.html).
goat anti-Sub P (1:500, Santa Cruz, Cat# sc-9758) (https://www.scbt.com/p/substance-p-antibody-n-18)
rabbit anti-PGP9.5 (1:1000, Millipore, Cat# AB1761-I) (https://www.emdmillipore.com/US/en/product/Anti-Protein-Gene-Product-9.5-Antibody,MM_NF-AB1761-I)
rabbit anti-Piezo1 (1:400, Novus Biologicals LLC, Cat# NBP1-78537) (https://www.novusbio.com/products/piezo1-antibody_nbp1-78537)
rabbit anti-p-CREB (1:500, Cell Signaling, Cat#) (https://www.cellsignal.com/products/primary-antibodies/phospho-creb-ser133-87g3-rabbit-mab/9198; western blot in the current study Figure S10b)
rabbit anti-p-Akt (1:500, Cell Signaling, Cat# 4046) (https://www.cellsignal.com/products/primary-antibodies/phospho-akt-ser473-d9e-xp-rabbit-mab/4060; western blot in the current study Figure S10a)molecular probes
Isolectin GS-IB4 (1:500, Molecular Probes, Cat# I21411) (https://www.thermofisher.com/order/catalog/product/I21411
Alexa 647-conjugated anti-mouse CD80 (1:200, Biolegend, Cat# 104718, Clone 16-10A1) (https://www.biolegend.com/en-us/products/alexa-fluor-647-anti-mouse-cd80-antibody-3119?GroupID=BLG6876)

Animals and other research organisms

Policy information about studies involving animals; ARRIVE guidelines recommended for reporting animal research, and Sex and Gender in Research

Laboratory animals

Male and female mice that were used to generate data were at 2-3-month old except being specifically indicated in each experiment. The parental mice were either purchased from The Jackson Laboratory (JAX) or obtained from other investigators. Standard

husbandry conditions with 12:12-h light cycles and free access to regular food/water were provided to each cage that housed 2–5 mice to ensure adequate social environment. All experimental protocols involving animal use were approved by Virginia Commonwealth University Institutional Animal Care and Use Committee (IACUC). Animal care was in accordance with the Association for Assessment and Accreditation of Laboratory Animal Care (AAALAC) guidelines.
Animals used are listed below:
Piezo2;GFP mice that were generated by crossing Piezo2-EGFP-IRES-Cre mice (Piezo2-Cre, JAX Stock # 027719, B6(SJL)) with EGFP::L10a mice (JAX Stock # 024750, B6)
Piezo2;hM3Dq mice that were generated by crossing Piezo2-Cre mice with RC::L-hM3Dq (JAX Stock # 026943, B6)
Wt;hM3Dq mice that were generated by crossing wildtype mice (C57BL/6) with RC::L-hM3Dq
Piezo2;ChR2 mice that were generated by either crossing Ai27D mice (JAX Stock # 012567, B6) or Ai32 mice (JAX Stock # 012569, B6)
Nav1.8;tdTomato;GCaMP mice that were generated by crossing Nav1.8-Cre+/+ mice (a line that was created by Dr. John Wood, Wolfson Inst. UK) with PC-G5-tdT mice (JAX Stock # 024477, B6)
Nav1.8;EGFP::L10a mice that were generated by crossing Nav1.8-Cre mice with EGFP::L10a mice
Piezo2;YFP mice and Nav1.8;YFP mice that were generated by crossing Ai32 mice with Piezo2-Cre or Nav1.9-Cre mice, respectively
Piezo2cKO mice that were generated by using Nav1.8-Cre+/+ mice and floxed Piezo2 (Piezo2fl/fl) mice (JAX Stock # 027720, B6(SJL))
Piezo2wt mice that were generated by crossing Nav1.8-Cre+/+ mice with wildtype mice
Csf1R;GFP mice that were generated to genetically label macrophages with GFP, which was produced by breeding Csf1r-Cre mice (JAX Stock # 029206, C57BL/6) with EGFP::L10a mice.

Piezo2-EGFP-IRES-Cre mice (Piezo2-Cre, JAX Stock # 027719)
EGFP::L10a mice (JAX Stock # 024750).
RC::L-hM3Dq (JAX Stock # 026943).
Ai27D mice (JAX Stock # 012567)
Ai32 mice (JAX Stock # 012569)
PC-G5-tdT mice (JAX Stock # 024477)
Piezo2 (Piezo2fl/fl) mice (JAX Stock # 027720)
Csf1r-Cre mice (JAX Stock # 029206)

Wild animals	Wild animals were not used in this study.
Reporting on sex	This study focused on sex-dependent differences. Male and female mice were used.
Field-collected samples	No field-collected samples were used in this study.
Ethics oversight	Institutional Animal Care and Use Committee (IACUC) at Virginia Commonwealth University

Note that full information on the approval of the study protocol must also be provided in the manuscript.

Flow Cytometry

Plots

Confirm that:

☒ The axis labels state the marker and fluorochrome used (e.g. CD4-FITC).

☒ The axis scales are clearly visible. Include numbers along axes only for bottom left plot of group (a 'group' is an analysis of identical markers).

☒ All plots are contour plots with outliers or pseudocolor plots.

☒ A numerical value for number of cells or percentage (with statistics) is provided.

Methodology

Sample preparation	Dorsal root ganglia (DRG) from mice were freshly dissected out and subject to enzymatic dissociation prior to cell sorting.
Instrument	BD Fortessa cell analyzer
Software	FlowJo
Cell population abundance	The total events were around 200-400k.
Gating strategy	The scatter plot was generated using SSC-A versus FSC-A, along with each fluorophore of interest versus FCS-A. The gating was determined by labeling live/dead populations in combination with primary antibody exclusion as negative control, as well as using single fluorophore of interest.

☒ Tick this box to confirm that a figure exemplifying the gating strategy is provided in the Supplementary Information.
